# Optical ‘magnetic mirror’ metasurfaces using interference between Fabry-Pérot cavity resonances in coaxial apertures

**DOI:** 10.1038/srep10297

**Published:** 2015-05-28

**Authors:** Ranjith Rajasekharan, Ann Roberts

**Affiliations:** 1 Department of Electrical and Electronic Engineering, The University of Melbourne, Victoria, 3010, Australia; 2 School of Physics, The University of Melbourne, Victoria, 3010, Australia

## Abstract

Here we propose and computationally demonstrate a quasi-planar metasurface consisting of arrays of pairs of concentric coaxial apertures in a metallic film. The structure relies on destructive interference between Fabry-Pérot modes excited in each aperture at resonance producing transmitted fields that interfere destructively leading to suppressed transmission. Conversely, we show that in the case of a perfect conductor, near-perfect, broadband reflection can be achieved with zero phase change in the electric field and a variation of 2π on passing through the coincident resonances. Extending the concept to shorter wavelengths, we show that mirrors exhibiting close to a 2π phase excursion, albeit with a reduction in the amplitude reflection coefficient at resonance and a lower Q, can be also achieved. Structures such as these can be used to enhance light-matter interactions at surfaces and act as high impedance ground planes for antenna applications.

There is considerable current interest in the utilization of the phenomenon of mode interference in nanophotonics to develop a range of ‘metasurfaces’^1^,^2^ that have the ability to control the properties of transmitted or reflected electromagnetic radiation. In particular, high impedance^3^,^4^ surfaces or optical ‘magnetic’ mirrors^5^,^6^,^7^,^8^ have attracted attention for their capacity to enhance light-matter interactions by tuning the phase relationship between the incident and reflected electric fields. In particular, by ensuring that the two fields are in phase, the total electric field near the metasurface can be maximised leading to enhanced light-matter interactions. Furthermore, the phase of either the transmitted or reflected field can be spatially modified to produce flat lensing^9^ or other waveshaping surfaces^10^.

These metasurfaces rely on the interference between two modes within the unit cell of the structure. Examples include the electric and magnetic dipole resonances of micron scale dielectric cubes^6^. Here we investigate a metasurface consisting of pairs of resonant apertures in a metal film where the thickness of the film plays a key role. Each unit cell consists of concentric coaxial apertures each of which exhibits resonances that can be geometrically tuned to spectrally overlap. In particular, we take advantage of the finite thickness of the metasurface to adjust the first-order Fabry-Pérot resonance of a larger aperture so that it overlaps with the zero-order resonance of a smaller aperture. In this case the transmission through the film is suppressed since the fields at the back surface of the film are out of phase with each other. On the other hand, the fields at the incident surface are in phase so electromagnetic waves are strongly reflected from the surface and there is zero phase shift between the incident and reflected plane waves. In the case of a surface composed of a perfect electric conductor (PEC), such a ‘mirror’ is near perfect. When the optical properties of metal in the visible region of the spectrum are taken into account, the reflection, although varying, is still relatively strong and the reflected field is in phase with the incident field at resonance.

The phenomenon demonstrated here relies on resonances of apertures in a metallic film. This topic has long attracted interest, whether in early investigations of the transmission of radio-frequency electromagnetic waves through apertures in a perfect conductor^11^,^12^, as elements in frequency-selective surfaces^13^,^14^,^15^,^16^ and, more recently, through their role in the phenomenon of enhanced transmission^17^. Both isolated apertures and holes in periodic arrangements have been investigated. In particular, some apertures, including narrow slots^18^ and coaxial apertures^16^,^19^,^20^,^21^,^22^,^23^,^24^,^25^,^26^,^27^, exhibit distinct localized resonances. The resonances of these apertures can be regarded as cavity modes with both a transverse and longitudinal dependence. Generally, the thickness of metal used in experiments, however, is so small, that the spectrum of longitudinal Fabry-Pérot resonances cannot be seen. As the thickness of the metal increases, the spectral separation between these modes decreases, the higher-order Fabry-Pérot resonances are red-shifted and it becomes possible to identify the influence of these higher order resonances^28^,^29^. Indeed if a unit cell contains two resonant apertures that can be independently tuned, it is possible to tune the interference between these modes, hence modifying the amplitude of the far-field transmission through, and reflection from, the film. The phase relationship between the modes on the reverse side of the film will depend on whether the resonances are both even order or both odd order vertical Fabry-Pérot resonances, or whether one is even and the other odd. In the latter case, we expect that the radiation from each aperture will interfere destructively leading to a *minimum* in transmission associated with the resonance. In the case of a PEC film, however, the absence of any loss mechanism, means that it would be expected that the reflectivity of the surface would remain high producing a resonant mirror.

Specifically, we here computationally investigate resonances of a hexagonal periodic arrangement of pairs of concentric coaxial apertures in a metallic film (Fig. 1). We consider separately the cases where the film is assumed to be, firstly, a perfect electric conductor (PEC) and, secondly, silver in the visible region of the electromagnetic spectrum. In the former case a modal method in the monomodal approximation is used, whereas a full-field Finite Element Method (FEM) solver is used to study apertures in silver films.

## Results and Discussion

**Figure 1:**
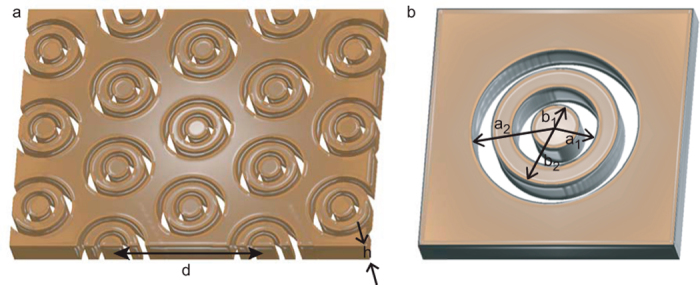
Schematic showing (**a**) a hexagonal array of pairs of concentric coaxial apertures in a metal film and (**b**) a pair of concentric coaxial apertures with parameters used to describe the geometry.

A metal film of thickness *h* is perforated with a hexagonal array (period *d*) of pairs of concentric annular apertures (Fig. 1). The smaller ring has outer and inner radii, *a*_1_ and *b*_1_, respectively, while the equivalent radii for the larger aperture are *a*_2_ and *b*_*2*_ (Fig. 1(b)). Single coaxial apertures in metallic films^16^,^30^,^31^ show strong dipolar plasmonic resonances associated with a TE_11_-like waveguide mode in the cavity. The optical performance of the structure investigated here involves the excitation of two largely decoupled modes: (i) a zeroth-order vertical Fabry-Pérot resonance of the fundamental transverse dipole mode of the smaller of the two apertures, and (ii) a first–order Fabry-Pérot resonance of the larger aperture that depends on both the transverse size of the aperture and the thickness of the metal. Both modes independently couple efficiently to a normally incident plane wave and have in-phase electric fields at the excitation surface. This leads to strong reflection where the reflected field is *in phase* with the incident field in contrast to a conventional mirror. On the reverse surface, however, the surface charges create oppositely directed fields that are approximately equal in magnitude leading to a minimum in the transmitted power. In addition, the symmetry of the structure is consistent with performance that is independent of the polarization of normally incident electromagnetic waves. This concept has similarities with previous work looking at the design of a metasurface, consisting of two orthogonal apertures, that behaves as a quarter-wave plate at a design wavelength^32^.

### Perfectly Electrically Conducting Metasurface

We first consider apertures in a thick PEC film using an electromagnetic modal method^13^. In the case of apertures in metals used at millimetre wavelengths or in the far-infrared, the assumption of PEC is in good agreement with experimental results. It has been shown, however, that some essential features of their behavior extend to much shorter wavelengths despite the effects of the finite-conductivity becoming apparent.

**Figure 2:**
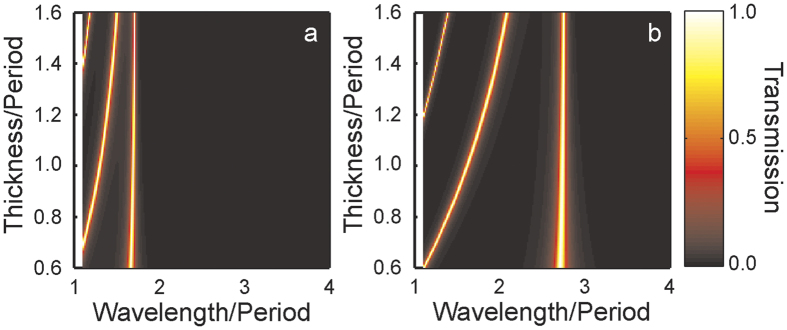
Transmission through hexagonal arrays of coaxial apertures in a PEC film calculated using the modal method in the monomodal approximation as a function of both wavelength and film thickness. In (**a**) the outer and inner radii are 0.3*d* and 0.25*d* respectively, while in (**b**) the corresponding figures are 0.45*d* and 0.43*d*.

To gain insight into the performance of the array of pairs of coaxial apertures, we first consider a hexagonal array of periodicity *d* of single annular apertures of outer radius, *a* and inner radius, *b*, in a free-standing PEC film of thickness *h.* Values for these parameters were selected to create distinct and well-separated resonances and ensure that only one mode (the coaxial waveguide TE_11_ mode) is propagating within the holes. The film is illuminated with a normally incident plane wave of wavelength 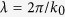, where *k*_*0*_ is the wavenumber. Figure 2 shows the transmission calculated using the modal method in the monomodal approximation through the two different arrays of apertures. In the case of Fig. 2(a), the outer radius of the ring is *a*_*1*_ = 0.3*d* and the inner radius *b*_*1*_ = 0.25*d*. In the case of Fig. 2(b), *a*_*2*_ = 0.45*d* and *b*_2_ = 0.43*d*. The transmission is shown as a function of both wavelength and the thickness of the film. Note that only wavelengths where there is only one propagating reflected and transmitted plane wave are considered.

It is apparent that, in the case of Fig. 2(a), there is a resonance at a wavelength of 1.67*d* that is independent of the thickness of the film. In the case of the larger coaxial aperture shown in Fig. 2(b), the longest wavelength resonance is at 2.7*d*. It is similarly robust to the thickness of the film. These are the zeroth order (*m* = 0) Fabry-Pérot resonances of the TE_11_ modes of the two cavities^16^. It is apparent, however, that there are resonances occurring at shorter wavelengths that depend on the film thickness. These are higher order (*m* > 0) vertical Fabry-Pérot resonances of the same transverse mode. Here we focus on the resonance shown in Fig. 2(b) that moves from a wavelength of approximately 1.1*d* to 2.1*d* as the thickness increases from 0.6*d* to 1.6*d*. At a thickness of 1.075*d*, this resonance coincides with the zeroth order Fabry-Pérot resonance of the smaller aperture. Note that since the film is assumed to be a PEC the absence of loss means that the peaks of 100% transmission are accompanied by troughs of 0% in the reflection spectra.

**Figure 3:**
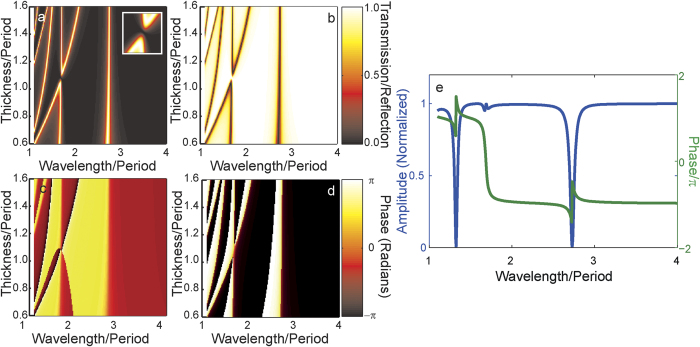
Transmission through and reflection from hexagonal arrays of pairs of coaxial apertures in a PEC film calculated using the monomodal approximation in the rigorous modal method as a function of both wavelength and film thickness. In (**a**), the transmission through an array of double concentric coaxial apertures with the dimensions of the apertures in Fig. 2 is shown (the inset shows transmission for thickness ranges from 1.005*d* to 1.145*d* and wavelengths from 1.58*d* to 1.78*d*) while (**b**) shows the reflection 1-T. Plots of the phase (wrapped from –π to π) of the transmitted (**c**) and reflected (**d**) fields are also shown. The amplitude (blue curve) and (unwrapped) phase (green curve) of the reflected field from an array of pairs of concentric coaxial apertures in a PEC film of thickness 1.075*d* is shown in (**e**).

Figure 3(a) shows the transmission through the equivalent array of the double coaxial structure with the same apertures using the modal method in the monomodal approximation. It is apparent that the transmission looks similar to a direct superposition of the plots of Fig. 2(a) and Fig. 2(b), with the exception of a distinct minimum in transmission where the first-order Fabry-Pérot resonance of the larger aperture crosses the zeroth order resonance of the smaller aperture. Since the two modes individually couple strongly to the transmitted plane wave, and the fields associated with the first-order Fabry-Pérot resonance of the larger aperture are out of phase with the zeroth-order Fabry-Pérot resonance of the smaller aperture at the lower surface of the cavity, these fields cancel and there is a distinct minimum in the transmitted field. Since the material is assumed lossless, this means that the reflection (Fig. 3(b)) is at a maximum. It is apparent from Fig. 3 that the structure behaves to a high degree as a broadband mirror at a thickness of 1.075*d* over a range of wavelengths from 1.3*d* to 2.7*d*.

As expected all resonances are accompanied by significant changes in phase of the transmitted (Fig. 3(c)) and reflected field (Fig. 3(d)). Fig. 3(e) shows the amplitude and (unwrapped) phase of the reflected field as a function of wavelength for a film thickness of 1.075*d*. In the case of the resonances of individual apertures (at wavelengths of 1.3*d* and 2.7*d*), the phase change in the reflected field is of the order of *π* as expected. As the wavelength passes through the resonance at 1.67*d*, however, the phase excursion as a function of wavelength is of the order of 2*π*. Furthermore, at a wavelength of 1.67*d* the phase of the reflected field is zero – it is in phase with the incident field.

### Finitely Conducting Metasurfaces

**Figure 4:**
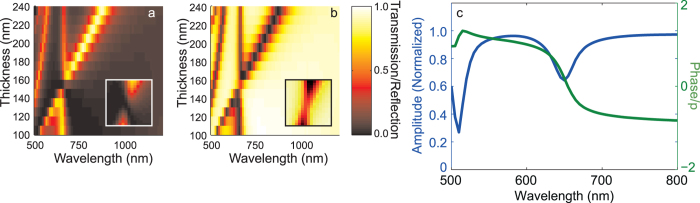
Transmission through (**a**) and reflection from (**b**) a hexagonal array (period 430 nm) of pairs of concentric coaxial apertures in a silver film of variable thickness. Data describing the geometry of the structure is given in the text. Values are normalized to the incident intensity. The insets in (**a**) and (**b**) highlight the region around the resonance for thicknesses between 141 and 159 nm and over a wavelength range from 590 nm to 740 nm. The amplitude (blue curve) and (unwrapped) phase (green curve) of the reflected field from an array of pairs of concentric coaxial apertures in a Ag film of thickness 147 nm is shown in (**c**).

We further demonstrate that this subwavelength interference effect can extend to real materials structured on the nanoscale that are resonant in the visible region of the electromagnetic spectrum. We use the Finite Element Method (FEM) in COMSOL Multiphysics with RF module (Version 4.4) with optical properties of silver taken from Johnson and Christie^33^. In Fig. 4, we show the transmission through a 430 nm period hexagonal free-standing array of concentric coaxial apertures where the thickness of the silver film varies. Here the smaller ring apertures have radii, *a*_*1*_ = 65 nm and *b*_*1*_ = 45 nm and the larger apertures have *a*_*2*_ = 180 nm and *b*_2_ = 160 nm. Again, the geometry was chosen to highlight the phenomenon under consideration, but remaining within the range of experimentally realizable structures. Note that the longest wavelength resonance of the structure investigated in Fig. 4 occurs at a wavelength longer than 1800 nm and is outside the range plotted. The existence of a resonance with an approximately linear dependence on film thickness (identified as a first order Fabry-Pérot resonance of the larger aperture) and an approximately thickness independent resonance (a zeroth order Fabry-Pérot resonance of the smaller aperture) is apparent. Furthermore, the destructive interference between the two modes at a wavelength near 655 nm for a thickness of 147 nm can be seen. In the case of real materials on resonance, increased losses in the Ag are expected and Fig. 4(b) shows strong reflection (albeit not 100%) in the reflection spectrum. Figure 4(c) shows the amplitude and phase of the field reflected from a film of thickness 147 nm. It is apparent that there is a near 2π phase excursion through the resonance near 655 nm and, although there is a decrease in the reflectance close to resonance due to enhanced absorption in the metal, that the reflectance remains high. Furthermore, at 655 nm, the reflected field is *in phase* with the incidence field, suggesting that this structure is behaving as a polarization-independent magnetic mirror at visible wavelengths.

**Figure 5:**
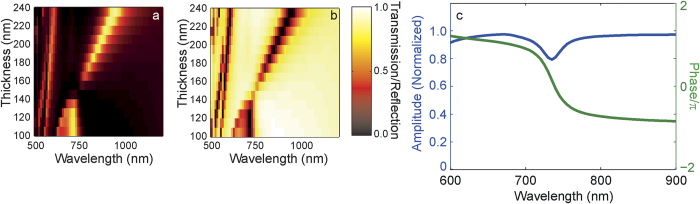
Normalized transmission (**a**) and reflection (**b**) through a silver film perforated with pairs of concentric coaxial apertures supported by a substrate with refractive index 1.5. Other parameters as for Fig. 4. The amplitude (blue curve) and (unwrapped) phase (green curve) of the reflected field for a film of thickness 145 nm is shown in (**c**).

To investigate the influence of a substrate required in fabricated structures, the calculations above were repeated in the presence of a substrate with refractive index 1.5 (Fig. 5). In this case the wavelength exhibiting the strongest transmission suppression and the zero phase shift of the reflected field has been red-shifted to 740 nm but the resonant thickness remains approximately 145 nm. Given the relatively thick metal film, the weak dependence on substrate index was anticipated^34^.

**Figure 6:**
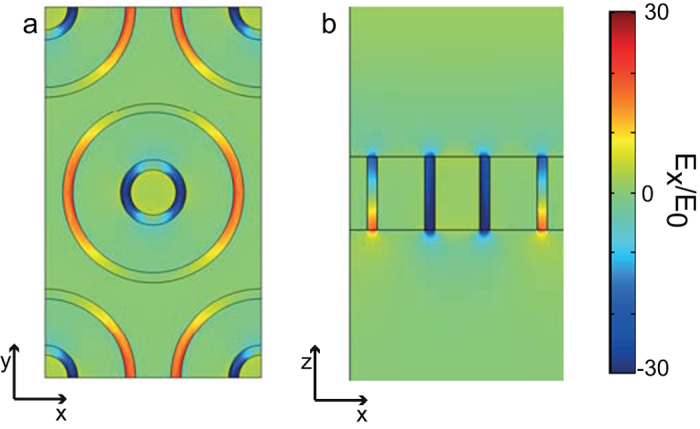
Fields in and around the apertures of the structure shown in Fig. 4 at resonance (655 nm) in a silver film of thickness 147 nm. Plots (**a**) shows the component of the electric field parallel to that of the incident field, *E*_*x*_, in a plane corresponding to the lower surface of the film, while (**b**) shows a vertical slice parallel to the plane of incidence through the center of a unit cell.

To illustrate the fact that this phenomenon is a result of interference between plane waves coupling to out-of-phase modes at the lower surface of the metal film, plots of the component of the electric field, *E*_*x*_, parallel to the incident field at *λ* = 655 nm and with *h* = 147 nm are shown in Fig. 6. Figure 6(a) shows a transverse slice at the lower surface of the film, while Fig. 6(b) shows a vertical slice through the center of the apertures parallel to the plane of incidence. Figure 6(a) exhibits the characteristic structure of the TE_11_ mode in each of the holes. In Fig. 6(b) the field shown is essentially a direct superposition of the two fields emerging from each aperture considered separately. The fact that the fields are in phase at the upper surface and out of phase at the lower surface is immediately apparent. Note that since the enhancements in fields within the apertures are not accompanied by a transmitted field, this phenomenon could also be useful in some sensing and imaging applications where low background, highly localized field enhancements are required.

**Figure 7:**
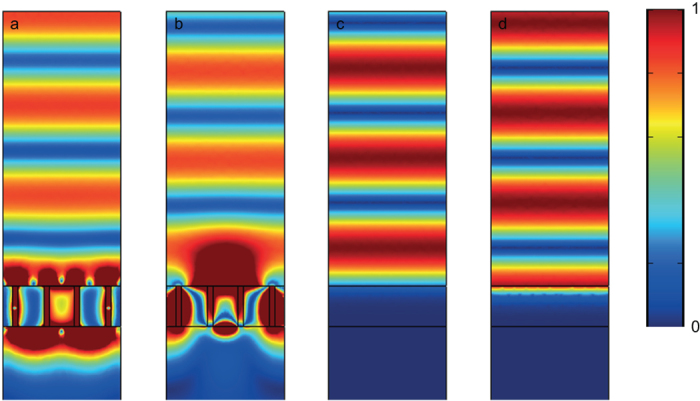
The magnitude of the total electric ((**a**) and (**c**)) and magnetic field ((**b**) and (**d**)) in the *x-*z plane above the free-standing metasurface in a Ag film of thickness 147 nm (**a**) and (**b**) and above a slab of Ag at 655 nm. For (**a**) and (**c**), the scale bar extends to twice the incident field amplitude and in (**b**) and (**d**), the range maximum is twice the amplitude of the incident magnetic field.

To further highlight the behavior of this structure as a magnetic mirror, the magnitude of the total electric field and total magnetic field above the structure at resonance is compared with that above a slab of Ag at 655 nm is shown in Fig. 7. It is clear that the mirror has shifted the phase of both the electric and magnetic fields considerably from what would be expected in the case of a conventional mirror.

The practical realisation of these remains an ongoing challenge, but structures with similar dimensions^35^ have been fabricated using focussed ion beam milling for use in the near-infrared. Reducing the size of the central aperture will reduce the thickness of the metal film required. This will, however, come at the cost of greater constraints on the transverse resolution required. The optimisation of the device in the context of a particular application, design wavelength and fabrication strategy will be required prior to an experimental demonstration of this device.

In conclusion, double coaxial apertures possess a rich array of modes that can be tuned to spectrally overlap. This permits the creation of a polarization-insensitive ‘magnetic’ mirror exhibiting 2π phase shift around resonance with a concept that can be utilized from millimeter waves down to the visible part of the spectrum. Furthermore, the additional degrees of freedom provide a versatile platform for studying plasmonic modes and developing new applications including as sensing elements, near-field imaging devices and resonant mirrors for the excitation of emitters.

## Methods

### Modal Method

The modal method involves writing the electric and magnetic fields within apertures in PEC films as a superposition of the relevant waveguide modes, while the fields in the superstrate and substrate are written as a discrete sum (in the case of periodic arrays) or an integral (in the case of isolated apertures) over plane waves. The amplitudes of the waveguide modes and the plane waves are found by imposing the relevant boundary conditions at the upper and lower surfaces of the film. This leads to a set of linear equations for the amplitudes of the waveguide modes that can be easily solved numerically. In the case where the PEC film is sufficiently thick and only one mode is propagating within the apertures, it is possible to obtain accurate calculations of far-field quantities using only this mode in calculations^13^,^36^. In this *monomodal* approximation relatively simple, closed form solutions for the amplitudes of the modes within the apertures can be written in terms of the aperture and film geometry, the admittance and propagation constant of the dominant TE_11_ mode of the coaxial waveguide mode, a sum over terms describing coupling between the modal fields and the diffracted set of propagating and evanescent plane waves and parameters describing the properties of the incident plane wave. Tests (not shown here) indicate excellent agreement between far-field values for the single coaxial aperture structure calculated using the monomodal approximation and a calculation using the modal method for arrays of coaxial apertures^16^ using 5TE and 5TM modes. Here we extend the monomodal approximation to the treatment of a periodic array of double coaxial apertures. Each aperture is assumed to support a single mode that can couple to the fields above and below the metal film and a closed form expression for the amplitude of the transmitted field found. The same strategy was used to investigate the performance of the quarter-wave plate of reference^32^. The modal method used here was implemented in IDL Version 8.3.

### Finite Element Method

We use the Finite Element Method (FEM) in COMSOL Multiphysics with RF module (Version 4.4) with optical properties of silver taken from Johnson and Christie^33^. The excitation field is a normally incident plane wave, port boundary conditions are used at the excitation and back surfaces of the modelled region, and periodic boundary conditions used on the sides.

## Additional Information

**How to cite this article**: Rajasekharan, R. and Roberts, A. Optical 'magnetic mirror' metasurfaces using interference between Fabry-Pérot cavity resonances in coaxial apertures. *Sci. Rep.* **5**, 10297; doi: 10.1038/srep10297 (2015).
